# EF-net: Accurate edge segmentation for segmenting COVID-19 lung infections from CT images

**DOI:** 10.1016/j.heliyon.2024.e40580

**Published:** 2024-11-20

**Authors:** Wenjin Zhong, Hanwen Zhang

**Affiliations:** University of New South Wales, Australia

## Abstract

Despite advances in modern medicine including the use of computed tomography for detecting COVID-19, precise identification and segmentation of lesions remain a significant challenge owing to indistinct boundaries and low degrees of contrast between infected and healthy lung tissues. This study introduces a novel model called the edge-based dual-parallel attention (EDA)-guided feature-filtering network (EF-Net), specifically designed to accurately segment the edges of COVID-19 lesions. The proposed model comprises two modules: an EDA module and a feature-filtering module (FFM). EDA efficiently extracts structural and textural features from low-level features, enabling the precise identification of lesion boundaries. FFM receives semantically rich features from a deep-level encoder and integrates features with abundant texture and contour information obtained from the EDA module. After filtering through a gating mechanism of the FFM, the EDA features are fused with deep-level features, yielding features rich in both semantic and textural information. Experiments demonstrate that our model outperforms existing models including Inf_Net, GFNet, and BSNet considering various metrics, offering better and clearer segmentation results, particularly for segmenting lesion edges. Moreover, superior performance on the three datasets is achieved, with dice coefficients of 98.1, 97.3, and 72.1 %.

## 1 Introduction

Medical imaging has significantly advanced due to deep learning (DL), demonstrating exceptional performance in areas such as segmentation, classification, object detection, and registration [[1], [2], [3], [4]]. Among these, convolutional neural networks (CNNs) have proven especially impactful, offering superior accuracy, efficiency, and reliability compared to traditional methods. Improved convolutional modules are more effective than other methods for tasks involving processing complex and varied patterns contained in medical images, such as distinguishing between healthy and diseased tissues [[5], [6], [7], [8]]. CNNs are adept at processing complex patterns in medical images, such as distinguishing between healthy and diseased tissues, by automatically identifying and focusing on relevant features like edges, textures, and shapes.

Integrating attention mechanisms with convolutional techniques has further enhanced model performance in medical imaging, particularly in lesion detection tasks. Attention mechanisms enable models to concentrate on key regions and features within images, addressing challenges posed by intricate anatomical structures [9,10]. This focus is particularly beneficial in identifying small but critical areas, such as lesions, which are often obscured by surrounding anatomical complexity.

During the global COVID-19 pandemic, deep learning (DL) methods were extensively utilized to develop effective models for diagnosing COVID-19 infections. Some notable approaches involved combining convolutional neural networks (CNNs) with attention mechanisms, which proved highly effective in identifying and segmenting COVID-19 lesion areas [[11], [12], [13]]. Attention mechanisms enhance feature weighting, enabling the network to focus on critical regions, such as abnormal lung areas, while addressing the considerable variability in the appearance and size of COVID-19 lesions. By selectively prioritizing relevant image features, these mechanisms significantly improve the accuracy of lesion detection and segmentation.

This study introduces a network structure that combines attention mechanisms with CNNs, especially focusing on edge features. The network achieves precise edge segmentation by effectively fusing edge and deep-level features. The contributions of this study are summarized as follows.

1. This study proposes a U-Net-based model for the precise segmentation of COVID-19 lesions using computed tomography (CT) images. The model comprises two main modules: an edge-based dual-parallel attention (EDA) module and a feature-filtering module (FFM). EDA aims to precisely segment edges in areas characterized by low-contrast levels and blurred boundaries, whereas FFM integrates low-level features with high-level features through two filtering gates. Additionally, we improve the parallel partial decoder [14], resulting in a deep-level feature aggregator (DFA) that aggregates features produced by a deep-level encoder and outputs predictions with lower resolutions. Finally, top-level decoders $D_{1}$ and $D_{2}$ use features derived from the EDA and DFA, which are full of edge and semantic information to generate a predicted outcome with accurately segmented edges, respectively.
2. EDA: We design the EDA module to better segment lesion edges. The module utilizes low-level features rich in edge information to precisely segment fuzzy and low-contrast boundaries contained in inputs. Features from different receptive fields are extracted using convolutional kernels of various scales and processed through two global attention branches to produce edge features and lesion edge prediction maps. The edge features are transmitted to FFM for feature fusion, and an edge-prediction map is used for edge prediction.
3. FFM: By introducing FFM, we fuse semantically rich features with edge features acquired from EDA. FFM contains two branches: with reverse attention (RA) [20] and complementary attention (CA). The filtering gates in both branches retain useful information obtained from the EDA and combine it with the semantic information derived from a deep-level encoder. Using this approach, we effectively fuse and utilize the obtained features.

In Section 2, we present the latest applications of DL in the field of lung CT image segmentation and delve into their advantages and limitations. Subsequently, we systematically describe our research methodology and corresponding solutions. Section 3 details the proposed network architecture and describes its key modules. Section 4 outlines the specific details of the experiments conducted using the proposed network to validate the effectiveness of the network structure and its core components. Finally, Section 5 summarizes the research findings and provides clear conclusions.

## 2 Related work

With the rise of DL in 2012, particularly the successful application of CNNs, medical image segmentation techniques have significantly developed. Considering the COVID-19 outbreak in 2019, DL methods have been extensively applied by medical personnel to analyze CT images. Numerous studies have used CNN models to detect and segment COVID-19 lesion areas in CT images [[14], [15], [16], [17], [18]].

Owing to blurred boundaries and low degrees of contrast between lesion areas and normal tissues in CT images, accurate segmentation of lesion areas remains a challenge. Numerous studies have proposed boundary-based and globally guided methods for precisely delineating lesion edges [19,20]. Edge guidance helps models clearly define boundaries between healthy and lesion tissues, enhancing the contrast between different areas in an image and allowing for easier identification and analysis of the areas of interest. Experiments [[14], [15], [16]] have demonstrated that edge guidance is crucial for accurately locating lesions and reducing classification errors, thereby producing more reliable and precise segmentation results and supporting the effective diagnosis of lesions. However, edge-guided algorithms are notably susceptible to variations in image content structures, particularly relevant given the significant differences between the shapes and sizes of COVID-19 lesion areas. Consequently, diverse structures of lesions influence edge-guided algorithms, often resulting in erroneous edge detection and imprecise segmentation.

Lesion areas often have irregular shapes and can be scattered in different lung locations, making networks prone to overlooking less obvious lesion areas. To focus on such inconspicuous areas, Chen et al. [21] developed an innovative approach known as RA for salient object detection. Their method shifted the focus of attention to less conspicuous areas, emphasizing the non-salient parts of images to better refine salient regions. The approach provided a new perspective and technique for improving salient target recognition accuracy. Although inverse attention aims to better detect salient targets by focusing on non-salient areas, the potential risk of neglecting certain salient areas exists, particularly when multiple small and large lesions are present in the input images, causing the model to overlook significant lesions.

To capture irregularly shaped lesion areas, multi-scale convolution has been widely used for capturing a range of features, from fine details to overall structures. By aggregating information at different scales, convolutional kernels of varying sizes improve the accuracy of COVID-19 lesion segmentation tasks. In experiments [[22], [23], [24]], multi-scale kernels extracted features from objects of different sizes in images, which is particularly beneficial for analyzing objects of significant size differences. However, as the number of parameters to be learned increases, the developed models face the risk of overfitting, particularly when the available training data are limited. For example, datasets comprising COVID-19 CT scans tend to be kept private to protect patient confidentiality.

Multi-level supervision allows models to analyze images at different scales or resolutions. Related studies [[14], [15], [16],^24^] have employed multi-level supervision approaches in COVID-19 lesion area segmentation tasks to capture fine-edge details and obtain more accurate segmentation results. Multi-level supervision captures detailed texture and edge information, whereas coarse-scale analysis captures global structural information. However, the features extracted at different scales can sometimes cause conflicts, resulting in the constructed model failing to learn effectively. Particularly, the issue is prominent in medical images because the differences between healthy and affected tissues can be subtle.

Moreover, Zhe et al. [25] noted higher resolutions and more computational resource requirements in low-level features than in deep features; however, limited performance improvements were recorded. Therefore, some methods such as the parallel partial decoder [14] and the aggregation modules in GFNet [15] only aggregate features from a deep-level encoder and output prediction images that are one-eighth the input size. However, the approach can produce suboptimal results because it discards texture-rich information contained in low-level features and uses only deep-level features for decoding. Although low-level features consume more computational resources, they retain texture and contour information that are crucial for segmenting lesion-edge areas.

To address the issue of inadequate segmentation of COVID-19 lesion areas [[14], [15], [16]], this study combines the advantages of the aforementioned modules to precisely locate and segment lesion parts, particularly by optimizing the process of segmenting lesion edges. Low-level features have higher resolutions and detailed texture information, but fewer semantics and more noise, whereas deep features possess semantic information, but have lower resolutions and poorer detail perception capabilities [26]. Therefore, we propose an EDA module that accepts low-level features, uses multi-scale convolution to extract features from different receptive fields, and further extracts the texture-rich features through a dual-branch attention structure to locate the edges of lesion areas. For semantically rich deep features, we design FFM using a branched structure, where one branch uses RA [21] to focus on small lesion areas, and the other focuses on significant lesion areas. Finally, the feature aggregation module aggregates the outputs derived from the encoders to obtain results at two different scales and uses multi-level supervision to enable the model to better learn finer global feature representations.

## 3 Proposed method

In this section, we provide a detailed introduction to the proposed edge parallel dual attention guide and feature filtering network (EF-Net) architecture, including its key modules and specific implementation details.

### 3.1 Overview of EF-Net

**Fig. 1:**
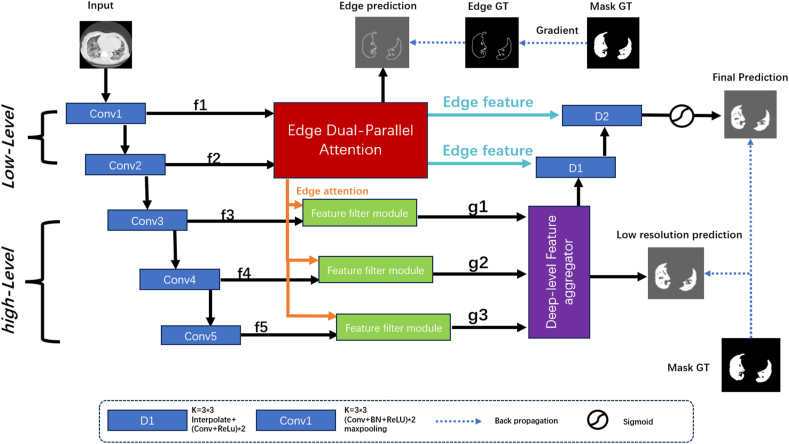
Architecture of our proposed EF-Net model.

The EF-Net framework shown in Fig. 1 has a U-shaped structure comprising five layers. Low-level features ($f_{1}$ and $f_{2}$) occupy the first two layers, while higher-level features are distributed across the third, fourth, and fifth layers ($f_{3}$, $f_{4}$, and $f_{5}$, respectively). The model comprises five encoders and two decoders, each maintaining a structure consistent with that of U-Net [27]. Additionally, we incorporate EDA to guide the edge feature extraction process, and then the features are passed on to FFM to merge them with semantic features derived from high-level encoders. Furthermore, a DFA module is employed to aggregate high-level features ($g_{1}$, $g_{2}$, $g_{3}$), generating low-resolution feature maps that are a quarter of the original image size. The final output is produced by another decoder $\left(D_{2}\right)$, and this output matches the input size. Further, we elaborate on the core modules of this network and the loss function employed.

### 3.2 EDA

Based on the concept of edge guidance, we considered low-level features possessing high-resolution rich textural information [22] and designed an EDA module that integrated the low-level features. The module received feature output from the first and second encoders and used a parallel dual-stream global attention mechanism to extract features that were full of edge information. The approach aided in the precise extraction of lesion area boundaries, addressed issues related to blurred boundary segmentation, and provided abundant textural information for subsequent modules.

**Fig. 2:**
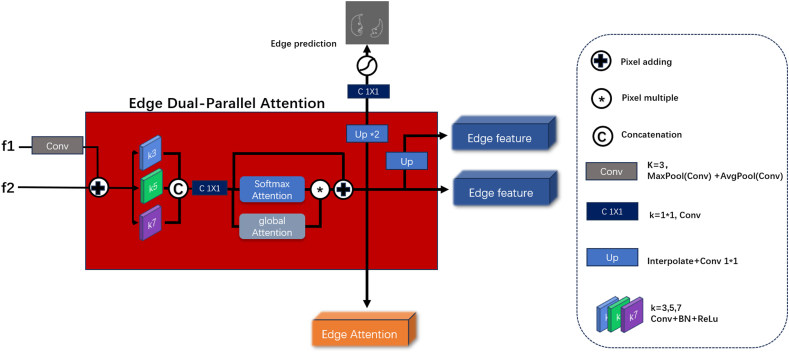
Illustration of the overall EDA module. Edge attention and edge features are obtained by FFM and decoders, respectively. Edge prediction is utilized for edge forecasting.

Initially, the features acquired from the first two encoders were processed through a convolution layer of 3 × 3 kernel size by standardizing the number of channels to 256. Subsequently, MaxPooling and AvgPooling were applied to $f_{1}$, followed by element-wise addition to obtain a feature map of the same size as that of $f_{2}$. Then, the processed $f_{1}$ was added in an element-wise manner to $f_{2}$, resulting in combined features. The process is illustrated in Fig. 2. MaxPooling and AvgPooling were simultaneously employed to retain as much of the original information as possible while reducing the image resolution.

To focus on texture and edge information under different receptive fields, we employed convolutional kernels of size 3 × 3, 5 × 5, and 7 × 7 for feature extraction. The use of multi-scale convolutional kernels enabled the extraction of features from varying-sized receptive fields comprising input features [23,24]. Particularly, the method was pertinent for COVID-19 lesion areas, which exhibit significant variations in size and shape. We employed three convolutional kernels with different receptive fields to extract texture and edge information from the input features and concatenated the features to form $f_{mix}$. The module is shown on the left side of Fig. 2.

**Fig. 3:**
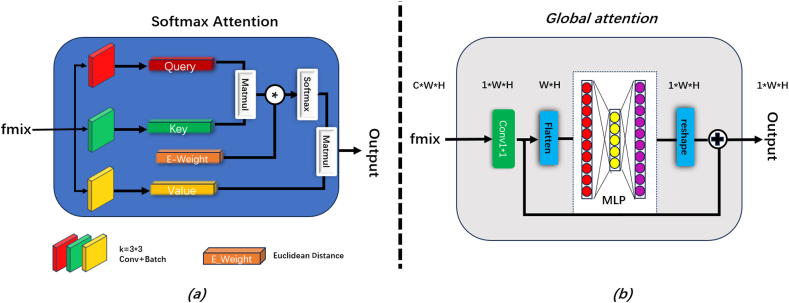
(a) Improved internal structure of Softmax attention. (b) Forward propagation process of Spatial attention.

After extracting the features with kernels of different scales, the resulting features $f_{mix}$ were fed into two parallel global attention modules, as illustrated in Fig. 3. Fig. 3(a) shows the Softmax attention mechanism [28], where three 3 × 3 convolutions were used to generate queries, keys, and values with unchanged channel numbers and kernel sizes, followed by a Softmax attention operation. The design enabled the model to amplify the weights of the pixels and areas that required attention from the queried pixel, thereby accurately concentrating on important image regions and providing improved segmentation performance.

Softmax attention was expressed as:

(1) $$\begin{array}{c} Attention\left(Q,K,V\right)=Softmax\left(\frac{QK^{T}}{\sqrt{d_{K}}}\right)V \end{array}$$

In the original study [28], $\sqrt{d_{K}}$ is scalar. We set $\sqrt{d_{K}}$ equal to the Euclidean distance [29] between the current query point ${Pix}_{q}$ and the queried point ${Pix}_{k}$. In our observations, when the pixels surrounding a queried pixel were all lesion pixels, the likelihood of the queried pixel being a lesion pixel was greater than that of a normal pixel. Based on such observations, we assigned a weight value to each pixel point, that is, the Euclidean distance between the two points. The closer a point was to ${Pix}_{q}$, the greater the weight assigned to it. Herein, Q, K, and V were obtained using Formula 2.

(2) $$\begin{array}{c} Q={Con}_{q}\left(f_{mix}\right)\;K={Con}_{k}\left(f_{mix}\right)\;V={Con}_{V}\left(f_{mix}\right) \end{array}$$

where ${Con}_{q}$, ${Con}_{k}$, and ${Con}_{v}$ are convolutions with kernel sizes of 3, stride = 1, and padding = 1, respectively. Further, Q, K, V, and $\sqrt{d_{K}}=E\_distance$ were incorporated into the attention formula. Our attention formula was expressed as:

(3) $$\begin{array}{c} Attention\left(Q,K,V\right)=Softmax\left(\frac{{Con}_{q}\left(f_{mix}\right){{Con}_{k}\left(f_{mix}\right)}^{T}}{\sqrt{{\sum_{x}^{w}\sum_{y}^{h}\left({Pix}_{q\left(x,y\right)}+{Pix}_{k\left(x,y\right)}\right)}^{2}}}\right){Con}_{V}\left(f_{mix}\right) \end{array}$$

The spatialattention(SpA) **branch** is shown in Fig. 3(b). SpA first used a 1 × 1 convolution for dimensionality reduction, setting the number of channels (C) to one. Then, the matrix was flattened to form a vector with H∗W dimensions, followed by two fully connected layers for global feature extraction; the matrix was finally reshaped to its original dimensions: 1∗H∗W. The **SpA** process was expressed as:

(4) $$\begin{array}{c} SpA=MLP\left(F\left(Conv\left(f_{mix}\right)\right)\right) \end{array}$$

To effectively merge the features extracted by the improved Softmax attention mechanism and SpA, we multiplied the output of the Softmax attention mechanism by the result of SpA. Subsequently, to further refine the detailed information focused by the two attention mechanisms, we performed an additional convolution operation on the merged features and introduced a shortcut connection. Thus, the details captured by the two attention mechanisms were effectively merged to form the final attention feature map.

Finally, to restore the feature map to its original image size, we performed two 3 × 3 deconvolution operations, thereby obtaining an edge prediction map. Specific calculation process for the EDA involved:

(5) $$\begin{array}{c} EdgeAttention=Dconv\left(SpA⊕Attention+f_{mix}\right) \end{array}$$

### 3.3 FFM

**Fig. 4:**
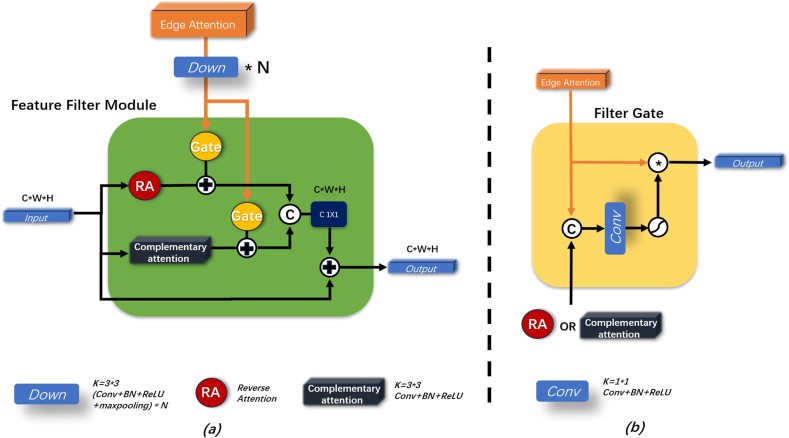
(a) Internal structure of FFM. (b) Structure of the filter gate and the feature filtering process.

Deep-level features contain rich semantic information [26] but suffer from low resolution, making it challenging for decoders to effectively extract edge and texture details by relying solely on such features. Therefore, FFM aimed to merge the deep features derived from $f_{3}$, $f_{4}$, and $f_{5}$ with the edge attention information provided by the EDA module. Through their fusion, we obtained features that were rich in semantic information and precise in terms of the textures and locations of the lesions. The structure of this module is illustrated in Fig. 4.

Experimental results indicated [20] that RA enabled the model to focus more on non-salient areas, significantly enhancing its ability to detect small lesion areas. The RA mechanism was expressed as:

(6) $$\begin{array}{c} RA=-1*Sigmoid\left(input\right)+1 \end{array}$$

However, the mechanism carried the potential risk of neglecting certain prominent areas. To compensate for the tendency of RA [34] to focus overly on non-salient parts and neglect prominent areas, we introduced CA to cover the significant regions that could have been missed by RA, as shown in Fig. 4(a).

To enable high-level features to incorporate edge attention information, we integrated two attention gates into the module processing the attention features acquired from the EDA through downsampling before entering the edge feature filtering gate. The structure of the feature-filtering gate is illustrated in Fig. 4(b), and was expressed as:

(7) $$\begin{array}{c} Gate=Sigmoid\left(Con\left(Concat\left(Input,EdA\right)\right)\right)⊙EdA \end{array}$$

The gate input was the output of either the RA or CA mechanism. Herein, Concat refers to concatenation.

Subsequently, the information derived from the EDA was filtered through the gate mechanism, retaining only the texture information required at the current level. Then, the information was merged with high-level features before being output to the DFA. Compared to traditional decoders that only acquired high-level features rich in semantics, our DFA obtained precise edge information after filtering. The improvement aided the DFA in preserving semantic information while restoring image edge details more accurately.

### 3.4 DFA

Other models, such as the parallel partial decoder [14] or the aggregation modules [15,16], often disregard low-level features that are full of texture and contour information when decoding features, relying solely on aggregating high-level features to identify lesion edges. However, the results obtained using such methods are often insufficiently accurate or clear. Contrarily, our proposed DFA aggregated features from $g_{1}$, $g_{2}$, and $g_{3}$, producing an output that was 1/8 the size of the original image. As $g_{1}$, $g_{2}$, and $g_{3}$ were already processed through the FFM, comprising the edge features required for decoding, the output retained both the semantic information and texture details of the EDA.

Furthermore, the deep-level features obtained by DFA were transmitted to decoders $D_{1}$ and $D_{2}$. Thus, $D_{1}$ and $D_{2}$ simultaneously accessed and interpreted the edge and semantic features derived and acquired from the EDA and DFA, respectively. The design enabled our model to precisely locate edges while considering the overall structure of the input image.

**Fig. 5:**
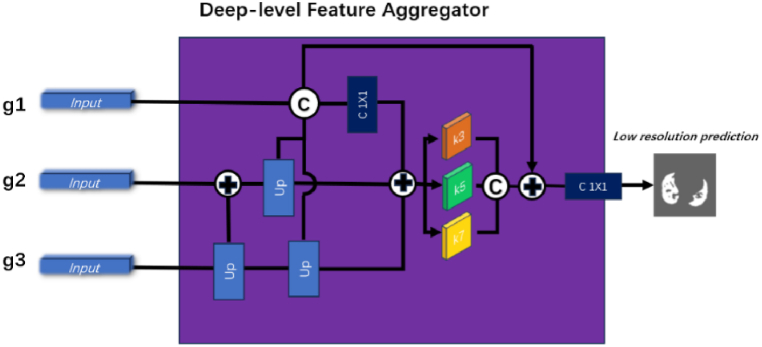
DFA aggregates features from three FFM modules: $g_{1}$, $g_{2}$, and $g_{3}$. It outputs a low-resolution prediction graph for backpropagation.

Therefore, our model made more comprehensive use of texture and contour information contained in low-level features and DFA assisted in aggregating features from three deep-level layers. Ultimately, features from the EDA and DFA were received using the two decoders, and then a result that matched the size of the input image was given as output, accurately segmenting the lesion edges while preserving the image details. The structure of the DFA is illustrated in Fig. 5.

### 3.5 Loss function

The designed loss function $L_{seg}$ involved a combination of a weighted intersection-over-union (IoU) loss and a weighted binary cross-entropy (BCE) loss that supervised all outputs, such that:

(8) $$\begin{array}{c} L_{seg}=L^{w}iou+L^{w}bce \end{array}$$

Unlike the standard IoU loss, which is commonly used in segmentation tasks, $L^{w}iou$ increased the weights of sample points derived from challenging pixels to emphasize their importance. In addition, compared with the standard BCE loss function, $L^{w}bce$ focused more on difficult pixel samples rather than assigning the same weight to all pixels. The weighted loss function was consistent with those considered in two prior research [30,31].

Therefore, the overall loss function was the sum of the edge loss, aggregator loss, and the loss induced by the final decoder output.

(9) $$\begin{array}{c} L_{total}=L_{seg}Edge+L_{seg}D2+\lambda L_{seg}Aggregator \end{array}$$

Herein, λ has an optimal value of 0.6 according to experimental tests.

## 4 Experiment

### 4.1 Experimental Environment and parameter settings

Our model was configured using PyTorch 1.10.0 and CUDA 11.8 frameworks and trained on a single NVIDIA RTX 4090 GPU with 24 GB of video memory. Preprocessing was not performed on the original images. The network was trained using an adaptive moment estimation (Adam) optimizer. We set the number of epochs to 300, batch size to four, and learning rate to 1e^−4^.

### 4.2 Evaluation metrics

We utilized five widely used metrics [31,32]: accuracy, mean IoU, Dice coefficient (*Dice*), sensitivity (*Sen.*), and specificity (*Spec.*) measures. In addition, we employed an objective detection metric: mean absolute error (MAE).

Metrics used for evaluation:

Accuracy (*Acc*) refers to the proportion of samples correctly predicted by the classifier out of the total number of samples. For this study, we defined accuracy as:

(10) $$\begin{array}{c} Acc=\frac{TP+TN}{TP+TN+FP+FN} \end{array}$$

Mean IoU refers to the percentage of overlap between the segmented mask and the corresponding label. For this study, we defined mean IoU as:

(11) $$\begin{array}{c} Mean\;IoU=\left(\frac{TP}{TP+FP+FN}\right) \end{array}$$

Dice Coefficient (*Dice*) is primarily used to calculate the similarity between two sets. For this study, we defined Dice as:

(12) $$\begin{array}{c} Dice=\frac{2\;*\;TP}{2*\;TP+FP+FN} \end{array}$$

Sensitivity (*Sen*.) represents the percentage of lung infections that are correctly segmented. For this study, we defined *Sen.* as:

(13) $$\begin{array}{c} Sen=\frac{TP}{TP+FN} \end{array}$$

Specificity (*Spec.*) represents the percentage of non-infected lung areas that are correctly segmented. For this study, we defined *Spec*. as:

(14) $$\begin{array}{c} Spec=\frac{TN}{TP+FP} \end{array}$$

MAE calculates the average of the absolute values of the prediction errors induced for each pixel. For this study, we defined MAE as:

(15) $$\begin{array}{c} MAE=\frac{1}{w*h}*\sum_{x}^{w}\sum_{y}^{h}\left|Pred\left(x,y\right)-GT\left(x,y\right)\right| \end{array}$$

### 4.3 COVID-19 CT dataset

We selected two publicly available datasets to validate the effectiveness of our model.

**COVID-19 CT Scan (CS)** [33]: This dataset comprised 3D CT scans from 20 patients diagnosed with COVID-19, along with expertly produced segmentations of their lungs and infections. The CT scan slices were of size 512 × 512 pixels relative to the x and y axes.

**COVID-19 CT Scan Lesion Segmentation Dataset (CLSD)**: This dataset combined COVID-19 lesion masks and their corresponding frames derived from three publicly available CT scan datasets [34,36], resulting in 2729 pairs of images and corresponding masks. All different types of lesions were mapped in white to maintain consistency across the datasets. The size of the image-mask pairs was 512 × 512.

**COVID-19 CT Image Segmentation Dataset (CISD)**: This dataset contained 829 slices and masks, each of size 512 × 512. The masks included four channels: 0 – “ground glass,” 1 – “consolidations,” 2 – “other lung areas,” and 3 – “background.” Additionally, the dataset features segmented axial volumetric CT scans acquired from nine patients and sourced from Radiopedia.

### 4.4 Experimental results and analysis

#### 4.4.1 Quantitative analysis

To comprehensively evaluate the segmentation performance of the proposed method with respect to the infected areas, we conducted in-depth comparative experiments using classic U-Net [26] and U-Net++ [37] models, and advanced models specifically designed for COVID-19 segmentation: Inf-Net [14], GFNet [15], and BSNet [16]. The quantitative analysis results obtained for the CS, CLSD, and CISD datasets are presented in Table 1, Table 2, Table 3, respectively.

**Table 1:** Performance data produced by each model in terms of each indicator on the CS dataset.

	Acc	IoU	Dice	Sen.	Spec.	MAE
U-Net	92.7	84.5	86.2	88.3	87.5	0.032
U-Net++	94.1	87.7	89.1	89.6	90.1	0.025
Inf_Net	95.6	89.3	90.6	93.3	93.4	0.023
GFNet	96.5	91.4	93.3	95.7	94.9	0.021
BS_Net	97.4	93.3	95.4	96.8	96.5	0.019
Ours	98.7	95.8	97.3	98.8	98.9	0.016

**Table 2:** Performance data produced by each model in terms of each indicator on CLSD.

	Acc	IoU	Dice	Sen.	Spec.	MAE
U-Net	93.2	82.9	85.1	86.1	87.8	0.041
U-Net++	94.6	85.1	88.3	89.6	89.3	0.037
Inf_Net	95.6	88.7	92.5	92.9	92.2	0.029
GFNet	97.3	91.6	94.9	95.3	95.4	0.025
BS_Net	97.7	92.3	95.3	96.2	96.5	0.023
Ours	98.9	94.1	97.1	98.1	98.8	0.018

**Table 3:** Performance data produced by each model in terms of each indicator on CISD.

	Acc	IoU	Dice	Sen.	Spec.	MAE
U-Net	91.7	59.5	72.6	69.6	94.6	0.011
U-Net++	93.4	61.3	74.3	73.2	94.5	0.011
Inf_Net	93.8	62.3	75.0	75.3	94.4	0.012
GFNet	93.6	61.9	74.5	74.4	94.4	0.012
BS_Net	93.9	63.3	75.7	77.0	94.6	0.010
Ours	95.4	70.6	79.3	80.1	95.7	0.008

The experimental findings indicated that while the performances of conventional medical segmentation models, such as U-Net [26] and U-Net++ [37] were the worst, the models specifically tailored for COVID-19 segmentation, such as Inf-Net [14], GFNet [15], and BSNet [16], exhibited notable performance improvements. The improvements are listed in Table 1, Table 2, Table 3. Notably, the proposed EF-Net model outperformed all other models across every evaluation metric. According to the metrics outlined in Section 4.2, EF-Net performed excellently on all the datasets. Thus, the exceptional performance of EF-Net on unfamiliar datasets was confirmed, along with its adaptability and broad applicability across various datasets. We observed a significant decline in the performance of all models on the CISD dataset. The reduction in effectiveness was attributed to the insufficient number of samples and the high noise level in the CT images, which increased the susceptibility of the models to noise interference. These conditions significantly affected the reliability and accuracy of the test models. Further analysis indicated that the limited available data led to overfitting. Despite the challenges posed by data limitations and significant noise, EF-Net outperformed the other methods. EF-Net exhibited superior noise resistance and generalization capabilities, making it exceptionally adept at accurately segmenting previously unseen data points during training. Thus, the robustness and adaptability of EF-Net in handling complex real-world imaging scenarios were demonstrated.

**Fig. 6:**
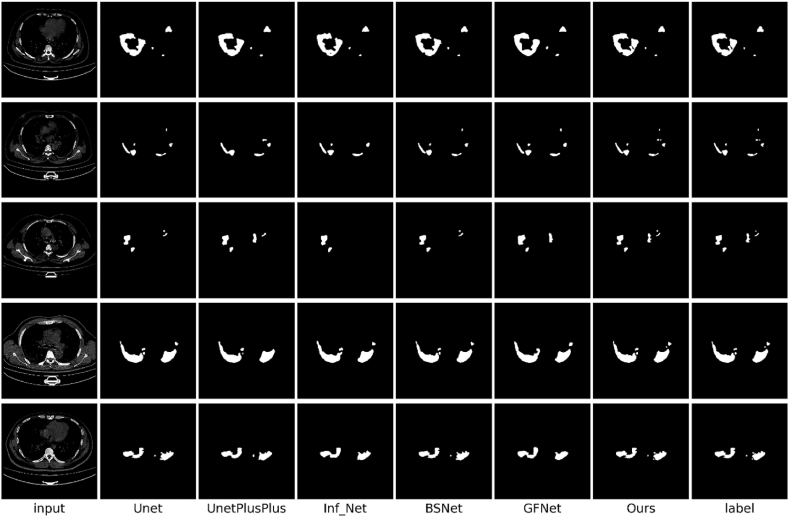
Segmentation results produced by different models on the CS dataset.

**Fig. 7:**
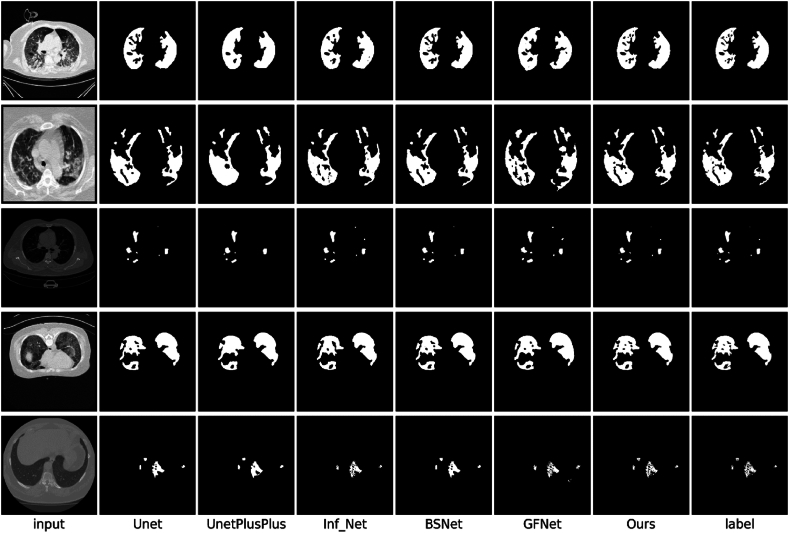
Segmentation results produced by different models on CLSD.

**Fig. 8:**
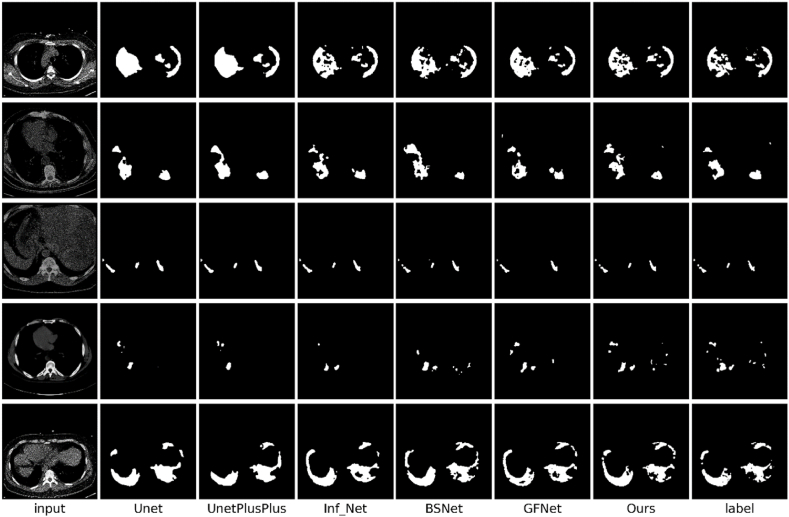
The segmentation results produced by different models on the CISD.

Comparisons between the segmentation outputs produced by our model and other models on the CS dataset, CLSD, and CISD, as depicted in Fig. 6, Fig. 7, Fig. 8, respectively, demonstrated the distinct advantages of EF-Net. The segmentation results of EF-Net were significantly closer to the ground truth, with markedly fewer errors and more precisely defined edge details. Conversely, U-Net [27] and U-Net++ [37] performed poorly owing to inadequate edge-guidance modules and single-scale supervision, leading to normal tissue misclassification and poor contour feature capture.

Inf-Net [19] used RA to focus on minor areas, potentially causing the model to overlook significant lesion regions. The decoding process employed a biased decoder that integrated only three deep features while discarding two shallow features rich in texture and contour information, resulting in less accurate edge segmentation. GFNet [15] and BSNet [16] made progress in edge segmentation through various edge module enhancement approaches, enhancing the utilization of shallow features and employing spatial attention to guide their models to focus on edge features. However, GFNet [15] and BSNet [16] lacked edge segmentation supervision, which led to imprecise lesion boundary segmentation.

EF-Net was unique because it employed a unique dual-stream global attention mechanism that effectively extracted comprehensive edge and texture information, delineated boundaries precisely, and used supervised guidance for edge segmentation. In addition, by utilizing RA [21], the proposed FFM focused on less prominent regions and balanced RA with CA to address areas potentially missed by RA, ensuring meticulous attention to smaller lesion areas without overlooking major regions. The integration of edge and informatic features within the FFM enabled the decoder to access rich textural and semantic information. EF-Net utilized DFA to merge all deep features and output them to a shallow decoder. Thus, deep semantic features and shallow contour-rich textures were given access, resulting in an output with precisely segmented edges.

### 4.5 Ablation experiment

Ablation experiments were conducted on the core components of EF-Net, including the EDA, FFM, and DFA, on the CS dataset and CLSD to validate the effectiveness of each module. The detailed experimental results are presented in Table 4, Table 5, Table 6.

**Table 4:** Results of model ablation experiments conducted on the CS dataset.

DFA	FFM	EDA	Acc	IoU	Dice	Sen.	Spec.	MAE
✔			93.8	86.5	88.8	88.9	89.7	0.027
	✔		95.8	89.4	90.6	93.7	93.5	0.023
		✔	96.5	91.5	93.7	94.9	95.7	0.022
✔	✔		93.7	90.6	92.4	93.1	94.2	0.024
✔		✔	96.9	92.7	95.1	95.7	95.9	0.021
	✔	✔	98.1	95.2	96.8	98.3	98.5	0.018
✔	✔	✔	98.7	95.8	97.3	98.8	98.9	0.016

**Table 5:** Results of model ablation experiments conducted on CLSD.

DFA	FFM	EDA	Acc	IoU	Dice	Sen.	Spec.	MAE
✔			93.9	84.9	86.9	87.1	88.5	0.038
	✔		95.3	88.9	92.0	92.4	93.5	0.029
		✔	96.2	90.9	93.2	94.3	94.4	0.028
✔	✔		95.4	89.3	93.2	93.4	93.5	0.029
✔		✔	97.6	91.8	95.6	96.2	96.8	0.024
	✔	✔	98.3	93.4	96.7	97.3	97.9	0.020
✔	✔	✔	98.9	94.1	97.1	98.1	98.8	0.018

**Table 6:** Results of model ablation experiments conducted on CISD.

DFA	FFM	EDA	Acc	IoU	Dice	Sen.	Spec.	MAE
✔			93.9	60.4	72.8	69.4	94.9	0.011
	✔		93.8	62.1	74.9	75.2	94.4	0.011
		✔	94.3	66.6	78.0	79.4	94.5	0.009
✔	✔		94.1	64.6	76.5	77.8	94.4	0.009
✔		✔	94.4	67.1	78.3	80.5	94.5	0.009
	✔	✔	95.0	69.9	82.1	81.4	94.9	0.008
✔	✔	✔	95.4	70.6	79.3	80.1	95.7	0.008

#### 4.5.1 Validating the effectiveness of DFA

In our experiment, we replaced the three deep-level decoders in U-Net [27] with the DFA module and conducted tests on the three datasets. The results clearly demonstrated that DFA, which aggregated deep-level features and output an additional low-resolution prediction map for multi-scale supervision, was crucial for achieving enhanced model performance. The DFA module significantly enhanced the ability of the network to capture detailed features, resulting in improved segmentation accuracy.

#### 4.5.2 Validating the effectiveness of FFM

Our experiments convincingly demonstrated the importance of FFM. As shown in Table 3, Table 4, Table 5, using FFM to fuse edge features with deep-level features significantly enhanced the segmentation performance of the model. FFM improved model performance by integrating shallow-layer texture features with deep-layer semantic features and selectively retaining pertinent features through a filtering process. The integration and filtering of the features resulted in more precise boundary detection and better overall segmentation, highlighting the value of incorporating edge information into deep feature maps.

#### 4.5.3 Validating the effectiveness of EDA

As shown in Table 3, Table 4, Table 5, the model with EDA outperformed the models with only DFA and FFM in terms of all evaluation metrics. The EDA module demonstrated remarkable robustness and versatility. The module effectively learned the boundary features of the target areas and precisely integrated them into the segmentation results, while organically merging them with the deep-level features. By organically merging with deep features, EDA enabled the decoder to extract richer features, indicating that fully utilizing texture information led to superior segmentation results. Fully utilizing low-level features resulted in superior segmentation outcomes.

#### 4.5.4 Combined effectiveness of DFA, FFM, and EDA

Finally, we conducted an experiment combining all three modules. The results indicated that when DFA, FFM, and EDA were used together, the model exhibited the best performance. Additionally, the combined use of EDA and FFM achieved excellent results, which were only slightly lower than the combined use of all three modules. Thus, we demonstrated the complementary nature of each module and its significant role in enhancing the overall performance of the model. The synergy among these modules contributed to the ability of the model to capture and integrate multi-scale, edge, and boundary features, leading to state-of-the-art segmentation performance.

### 4.6 Experiment on the effectiveness of simultaneously using CA and RA

In this section, we describe the experiments conducted to demonstrate the effectiveness of simultaneously using CA and RA [21] for assigning balanced attention to multiple aspects.

The experimental results (Table 7, Table 8, Table 9) indicated that using RA alone outperformed using CA alone, as RA focused on small lesion areas, thereby improving the model performance. To prevent the RA module from overly focusing on minor areas and neglecting significant regions, we employed a parallel approach that integrated both CA and RA. The approach allowed RA to focus on non-salient areas, whereas CA focused on significant lesion regions. The experiments demonstrated that the proposed method was superior to using either the RA or the CA module alone.

**Table 7:** Results of ablation experiments concerning RA and CA in FFM, conducted on the CS dataset.

	Acc	IoU	Dice	Sen.	Spec.	MAE
Feature gate (CA)	98.1	94.7	96.4	97.6	97.8	0.018
Feature gate (RA)	98.4	95.3	96.8	98.3	98.4	0.017
Feature gate (both)	98.7	95.8	97.3	98.8	98.9	0.016

**Table 8:** Results of ablation experiments concerning RA and CA in FFM, conducted on CLSD.

	Acc	IoU	Dice	Sen.	Spec.	MAE
Feature gate (CA)	98.3	93.1	95.6	96.7	97.4	0.021
Feature gate (RA)	98.6	93.7	96.4	97.6	98.3	0.020
Feature gate (both)	98.9	94.1	97.1	98.1	98.8	0.018

**Table 9:** Results of ablation experiments concerning RA and CA in FFM, conducted on CISD.

	Acc	IoU	Dice	Sen.	Spec.	MAE
Feature gate (CA)	95.3	70.3	79.2	80.1	95.2	0.008
Feature gate (RA)	95.4	70.5	79.3	80.1	95.7	0.008
Feature gate (both)	95.4	70.6	79.3	80.1	95.7	0.008

### 4.7 Validating the versatility of the modules possessed by EF-Net

To validate the versatility of the EF-Net model, we conducted a series of experiments integrating its core components into Inf-Net [26] and observed a significant performance improvement (specific data are presented in Table 10, Table 11, Table 12).

**Table 10:** Results obtained on the CS dataset by replacing the Inf-Net components with the components presented in this paper.

Inf_Net	DFA	FFM	EDA	Acc	IoU	Dice	Sen.	Spec.	MAE
✔				95.6	89.3	90.6	92.3	92.4	0.023
✔	✔			96.0	90.4	91.2	93.1	93.1	0.021
✔		✔		96.2	90.8	92.1	94.7	94.3	0.021
✔			✔	96.9	92.1	95.8	96.2	97.3	0.020
✔	✔	✔	✔	97.8	94.2	96.3	97.8	97.7	0.018

**Table 11:** Results obtained on CLSD by replacing the Inf-Net components with the components presented in this paper.

Inf_Net	DFA	FFM	EDA	Acc	IoU	Dice	Sen	Spec	MAE
✔				95.6	88.7	92.5	92.9	92.2	0.029
✔	✔			95.9	89.5	93.4	93.8	93.4	0.021
✔		✔		96.2	89.9	93.5	93.9	93.6	0.021
✔			✔	97.3	92.1	95.0	95.7	96.4	0.020
✔	✔	✔	✔	98.4	93.7	96.8	97.9	98.2	0.018

**Table 12:** Results obtained on CLSD by replacing the Inf-Net components with the components presented in this paper.

Inf_Net	DFA	FFM	EDA	Acc	IoU	Dice	Sen	Spec	MAE
✔				93.8	62.3	75.0	75.3	94.4	0.012
✔	✔			93.8	62.7	75.2	76.2	94.5	0.012
✔		✔		93.9	63.2	75.6	77.0	94.6	0.012
✔			✔	94.5	67.7	76.5	78.2	94.9	0.010
✔	✔	✔	✔	95.3	70.1	78.8	79.6	95.7	0.008

**Fig. 9:**
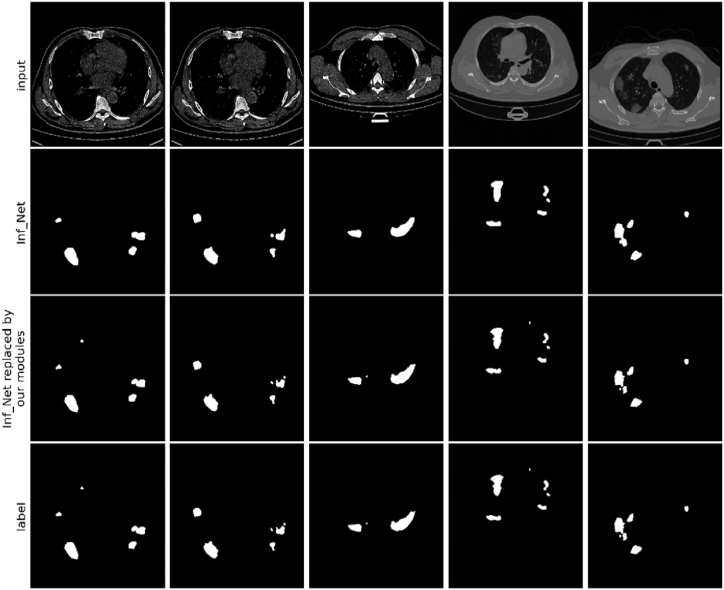
Results produced by Inf-Net before and after replacing its modules with our modules.

In our experiments, we replaced DDP, RA, and EG modules in Inf-Net [14] with DFA, FFM, and EDA modules, respectively. The results demonstrated that the modified model outperformed the original model in terms of various evaluation metrics, particularly when replacing the corresponding modules of Inf-Net [14] with all three modules from our study, yielding the best experimental results. Thus, our findings proved the effectiveness and versatility of the EF-Net modules. Fig. 9 shows the corresponding visualization results, further corroborating our conclusions.

### 4.8 Visualization of edge attention heatmaps

**Fig. 10:**
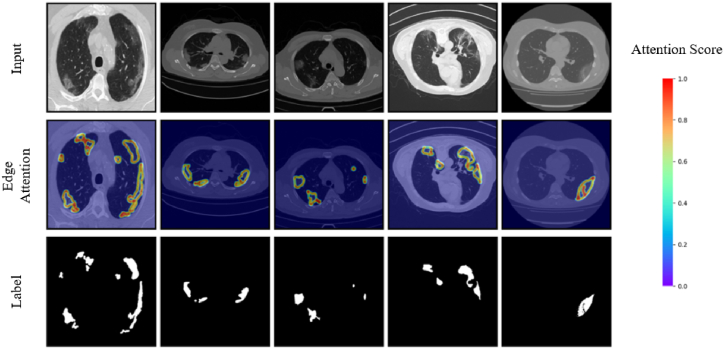
After the input CT image is passed through the EDA module, the output edge features are visualized as a heatmap. The redder a color, the more attention the corresponding region receives. (For interpretation of the references to color in this figure legend, the reader is referred to the Web version of this article.)

We extracted the features from the EDA module and visualized them using heat maps (Fig. 10) to provide an intuitive presentation. Through visualization, our EDA module evidently provided exceptional focusing capabilities, accurately targeting and concentrating on the edge and contour details of the lesion areas. The depth of color in the heatmap intuitively reflected the degree of attention given by the EDA module; the redder the area, the greater the level of attention given to that area by the EDA module.

### 4.9 Limitations and Future work

Despite the superior performance achieved by EF-Net on all datasets compared with those of existing methods, the training and testing speeds were slow in the experiments, which was attributed to the increased computational demands imposed by the attention mechanisms it employed. Owing to the time complexity of computing attention for each pixel against all other pixels being ${O_{\left(HW\right)}}^{2}$, which increases exponentially with the size of the input image, some existing medical models have improved their attention mechanisms to reduce their computational complexity. For instance, a medical transformer [38] calculates attention solely across rows and columns of pixels, thereby reducing the computational complexity to $O_{\left(HW\right)}$. Furthermore, BiFormer [39] utilizes a dynamic sparse attention mechanism that decreases the complexity of ${O_{\left(HW\right)}}^{\frac{4}{3}}$. Therefore, we intend to further refine the attention mechanism of the proposed model to maintain its optimal performance, while minimizing the required complexity. In addition, the edge segmentation strategy highlighted in this paper is vital for precisely delineating lesion boundaries. Future research should investigate the integration of self-attention mechanisms with edge detection technologies to segment infected areas in images more accurately. For example, implementing advanced edge-enhancement techniques can enable a model to better comprehend edge information, thereby enhancing its segmentation accuracy. We plan to continue optimizing EF-Net and combine it with more advanced modules to further improve its edge guidance and fusion modules, and optimize its computational efficiency.

## 5 Conclusion

This paper presents a novel regional segmentation network framework, EF-Net, specifically designed for COVID-19 pulmonary CT images. By incorporating EDA and FFM, EF-Net effectively captured information from areas in the input CT images with blurred boundaries, significantly enhancing the precision of boundary segmentation. The self-attention mechanism used in the EF-Net model demonstrated strong performance in the experiments, particularly in terms of addressing the global dependencies contained in large images. Additionally, a feature fusion strategy was utilized to comprehensively process the features of the encoder and further optimize the model output. Using two existing datasets, we validated the learning capabilities, generalizability, and robustness of EF-Net. The experimental results demonstrated that EF-Net surpasses previous models in terms of the accuracy and clarity of its segmentation results, particularly for edge-segmentation tasks. This was largely due to the robustness and versatility of the EDA module in EF-Net, which precisely learned and identified the boundary features of the target areas, including small and complex regions, and effectively applied these features to the segmentation results. Furthermore, we confirmed that integrating CA alongside RA [21] into the FFM enhances the expressiveness of the model, while simultaneously focusing on both micro- and macro-areas. Similarly, substituting each module in EF-Net with the corresponding modules in Inf-Net yields superior results for both datasets. This suggests that the modules contained in EF-Net have the potential to enhance the performance of other models. Future research will focus on optimizing the self-attention mechanism to maintain its performance while reducing its computational complexity. Moreover, improvements concerning the combination of self-attention mechanisms with edge-detection technologies will be implemented to segment lesion areas in medical images more accurately. This strategy is not only effective for segmenting COVID-19 CT lesion areas but is also suitable for other types of medical image segmentation tasks. Therefore, we believe that EF-Net has vast development potential and applicability to image segmentation tasks with vague, low-contrast, and challenging-to-segment boundaries, such as brain tumor detection, polyp detection under colonoscopy, cell segmentation, and other areas of image segmentation. EF-Net holds promise as a powerful tool that can assist medical professionals in performing medical image segmentation. For example, the heatmaps generated by the attention mechanism provide an interpretable basis for decision-making during image segmentation, which will help increase medical professionals' trust in these automated tools and reduce their workloads.

## CRediT authorship contribution statement

**Wenjin Zhong:** Writing – review & editing, Writing – original draft, Visualization, Validation, Supervision, Project administration, Methodology, Data curation, Conceptualization. **Hanwen Zhang:** Writing – review & editing, Writing – original draft, Visualization, Formal analysis, Data curation.

## 6 Ethics and Consent

Two publicly available datasets were used in this paper.

**CS** [38]: This dataset consisted of 3D CT scans acquired from 20 patients diagnosed with COVID-19, along with expertly produced segmentations of their lungs and infections. The CT scan slices had sizes of 512 × 512 with respect to the x- and y-axes.

Link: https://www.kaggle.com/datasets/andrewmvd/covid19-ct-scans.

Data sources.

[1] Paiva, O., 2020. CORONACASES.ORG: Helping Radiologists To Help People In More Than 100 countries | Coronavirus Cases. [Online] Coronacases.org. Available at: <link> [Accessed March 20, 2020].
[2] Glick, Y., 2020. Viewing Playlist: COVID-19 Pneumonia | Radiopaedia.Org. [online] Radiopaedia.org. Available at: <link> [Accessed April 20, 2020].

Expert Annotations.

[3] Ma Jun, Ge Cheng, Wang Yixin, An Xingle, Gao Jiantao, Yu Ziqi, He Jian. (2020). COVID-19 CT Lung and Infection Segmentation Dataset (Version Verson 1.0) [Data set]. Zenodo. DOI

**CLSD:** This dataset combines COVID-19 lesion masks and their corresponding frames derived from three publicly available CT scan datasets [[34], [35], [36]], resulting in 2729 pairs of images and their corresponding masks. All different types of lesions were mapped in white to maintain consistency across the datasets. The size of each image-mask pair was 512 × 512.

Link: https://www.kaggle.com/datasets/maedemaftouni/covid19-ct-scan-lesion-segmentation-dataset.

## 7 Data and code Availability

The code data have been deposited in the Github: https://github.com/ZJohnWenjin/EF-Net-covid-19-lesion-segmentation.git with name of repository EF-Net-covid-19-lesion-segmentation.

## Declaration of competing interest

The authors declare that they have no known competing financial interests or personal relationships that could have appeared to influence the work reported in this paper.
